# Whole-genome sequence of *Xanthomonas arboricola* pv. *pruni* strain 9-4 causing peach bacterial spot in China

**DOI:** 10.1186/s12863-025-01377-4

**Published:** 2025-10-29

**Authors:** Jiayu Yuan, Chun Yan, Junnan Wang, Zhaolin Ji, Lina Yang

**Affiliations:** College of Plant Protection, Yangzhou University, Yangzhou, Jiangsu 225009 China

**Keywords:** *Xanthomonas arboricola* pv. *pruni*, Genome assembly, Genome annotation, peach, Bacterial spot

## Abstract

**Objective:**

Peach bacterial spot, caused by *Xanthomonas arboricola* pv. *pruni* (Xap), poses a significant threat to the global peach industry. Studies have indicated that 41 Xap strains isolated from various peach cultivars in China exhibit moderate genetic differentiation, clear specificity to peach cultivars, and limited geographical differentiation. The genomic information of 36 Xap strains isolated from different countries has been deposited to the NCBI genome database; However, complete genome sequences for Xap strains identified in China remain lacking. In this study, the whole genome of the Xap 9-4 strain, isolated from diseased peach leaves in China, was assembled and annotated.

**Data description:**

The genomic data of the Xap 9-4 strain was sequenced using the Illumina and PacBio platforms. The whole genome of Xap 9-4 consisted of a 5,174,371 bp circular chromosome and a 41,329 bp circular plasmid, encoding 4,660 protein-coding genes, 201 ncRNAs, 643 repeat regions and 7 prophages. Approximately 94.82% of the proteins were annotated. Phylogenic analysis revealed minimal genetic variation among Xap strains isolated from different countries. These findings will expand the genomic resources for Xap and provide a foundation for further studies on genetic diversity and pathogen-host interaction mechanisms.

## Objective

Peach (*Prunus persica* (L.) Batsch) is an important economically cultivated fruit trees and is popular to consumers due to its delicious flavor and high nutritional value [1]. Peach bacterial spot, caused by *Xanthomonas arboricola* pv. *pruni* (Xap), is one of the most serious bacterial diseases affecting peach trees worldwide and lead to devastating losses of the peach industry [2, 3]. It not only causes bacterial spot on peach leaves and fruits, but also induces stem canker [3]. The disease was first described in North America in 1903, and further observed in Europe, Africa, Oceania and Asia [4]. Additionally, Xap could also infect the most stone fruits (nectarine, plum, apricot, cherry, and almond) [5].

In the past ten years, bacterial spot has emerged as an epidemic disease in major peach-producing areas of China. Studies have shown that 41 Xap strains isolated from various peach cultivars in China display moderate genetic differentiation, along with distinct host cultivar specificity but weak geographical differentiation [6]. The whole genome sequences of 36 Xap strains isolated from different countries have been deposited in the NCBI Genome database [7, 8]. However, the Xap strains identified in China still lack complete genome sequences. To expand the genomic resources for further exploration of genetic diversity and pathogen-host interaction mechanism, we sequenced the pathogenic Xap strain 9-4, which was isolated from diseased peach leaves in China.

## Data description

The Xap 9-4 strain was originally isolated from peach leaves within typical bacterial spot symptoms in Wafangdian city, Liaoning province, China (39.627114°N, 121.979603°E) in 2015. A single colony of the Xap 9-4 strain was grown overnight in nutrient broth (NB) media using a shaker at 28℃ and 200 rpm. Genomic DNA was extracted using the FastPure Bacterial DNA Isolation Mini Kit (DC103, Vazyme, Nanjing, China). The concentration of genomic DNA was measured using a Qubit fluorometer (Invitrogen), and the integrity and purity were assessed by 1% agarose gel electrophoresis. 

The Xap 9-4 genome was sequenced using the Illumina HiSeq 4000 (second-generation sequencing) and the PacBio RS II (third-generation sequencing) platform at the Beijing Genomics Institute (BGI, Shenzhen, China). Draft genomic unitigs, which represent unambiguous groups of fragments, were assembled using the Celera Assembler (v8.3) based on a high-quality corrected circular consensus sequence subreads set. To improve the accuracy of the genome sequences, GATK (v1.6-13) was used to make single-base corrections. There were two contigs, and the complete genome of the Xap 9-4 strain was 5,174,371 bp with a GC content of 65.37%, consisting of one chromosome (5,133,042 bp in length with a GC content of 65.39%) and one plasmid (41,329 bp in length within a GC content of 62.73%) (Data file 1, Table 1) [9]. As shown in Data file 2, the genomic assembly sequences were submitted to the NCBI GenBank Database with accession number CP182134 and CP182135 under bioproject number PRJNA1148038, representing the chromosome and plasmid of strain Xap 9-4 [10]. Moreover, the whole genomic sequencing coverage rate was 100% (Data file 8, Table 1) [9].

Gene prediction was conducted on the 9-4 genome assembly using Glimmer (v3.02) with Hidden Markov models [11]. tRNA, rRNA, and sRNAs were identified using tRNAscan-SE (v1.3.1) [12], RNAmmer (v1.2) [13], and Rfam database (v9.1) [14]. Tandem repeats annotation was performed using the Tandem Repeat Finder (v4.04), and minisatellite DNA and microsatellite DNA identified based on the number and length of repeat units. Prophage regions were predicted using the PHAST (v2013.03.20) [15]. The 9-4 genome contains 4,660 protein-coding genes, 201 ncRNAs, 643 repeat regions, 7 prophages (Data file 1, Table 1) [9]. Circular representations of the chromosome and plasmid were presented in Data file 3 [9].

Seven databases, which are KEGG, COG, NR, Swiss-Prot, GO, TrEMBL, and EggNOG, were used for general function annotation. Four databases were employed for pathogenicity and drug resistance analysis. Virulence factors and resistance gene were identified based on the core dataset in VFDB and ARDB databases; two additional databases used were PHI and CAZy. Type III secretion system effector proteins were detected using EffectiveT3. These results are presented in Data file 1 and Data file 3. Protein annotations, CDS sequences, amino acids sequences, and predicted Type III effectors of the Xap 9-4 strain were provided in Supplementary Data file 4–7 [9]. We further blasted the type III effectors with other *Xanthomonas* species (https://euroxanth.ipn.pt/bacteria:t3e:t3e). Found there were 21 T3SS effectors, and no transcription activator-like (TAL) effectors (homologues of the avrBs3) in Xap 9-4 strain (Data file 9, Table 1) [9]. In addition, the phylogenetic analysis found that there was minimal genetic variation among Xap strains isolated from different countries (Data file 10, Table 1) [9].

In conclusion, this study presented the complete genomic information of the Xap 9-4 strain causing peach bacterial spot on leaves in China. The availability of these data provides a solid foundation for investigating the genetic diversity and pathogen-host interaction mechanisms.

**Table 1:** Overview of data files/data sets

Label	Name of data file/data set	File types (File extension)	Data repository and identifier (DOI or accession number)
Data file 1	Genomic analysis, BGISEQ statistics, PacBio Reads statistics, k-mer statistics, genomic assembly statistics, genomic feature, ncRNA statistics, repeat statistics, prophage statistics and Genomic annotation statistics of *Xanthomonas arboricola* pv. *pruni* strain 9-4	Word file (.docx)	Figshare (10.6084/m9.figshare.29573585.v1) [9]
Data file 2	Genome assembly of *Xanthomonas arboricola* pv. *pruni* strain 9-4	Fasta file (.fasta)	GenBank (http://identifiers.org/bioproject:PRJNA1148038) [10]
Data file 3	Circular representation of genome and plasmid, gene length distribution, COG, GO and KEGG annotation of *Xanthomonas arboricola* pv. *pruni* strain 9-4	Word file (.docx)	Figshare (10.6084/m9.figshare.29573585.v1) [9]
Data file 4	Protein annotation of *Xanthomonas arboricola* pv. *pruni* strain 9-4	Excel file (.xlsx)	Figshare (10.6084/m9.figshare.29573585.v1) [9]
Data file 5	CDS sequence of *Xanthomonas arboricola* pv. *pruni* strain 9-4	Fasta file (.fasta)	Figshare (10.6084/m9.figshare.29573585.v1) [9]
Data file 6	Amino acids sequence of *Xanthomonas arboricola* pv. *pruni* strain 9-4	Fasta file (.fasta)	Figshare (10.6084/m9.figshare.29573585.v1) [9]
Data file 7	Predicted type III effectors in *Xanthomonas arboricola* pv. *pruni* strain 9-4	Excel file (.xlsx)	Figshare (10.6084/m9.figshare.29573585.v1) [9]
Data file 8	Coverage depth of *Xanthomonas arboricola* pv. *pruni* strain 9-4 genome.	Word file (.docx)	Figshare (10.6084/m9.figshare.29573585.v1) [9]
Data file 9	Comparation of type III effectors in Xap 9-4 and other *Xanthomonas* strains.	PDF file (.pdf)	Figshare (10.6084/m9.figshare.29573585.v1) [9]
Data file 10	Phylogenetic analysis of strain 9-4 among other Xap strains based on *rsmB*, *ropB* and *gyrB* consecutive gene sequences	Picture file (.tif)	Figshare (10.6084/m9.figshare.29573585.v1) [9]

## Limitations

This Data Note presents the complete genomic information of the Xap 9-4 strain, based on data obtained from both the PacBio and Illumina sequencing platform. No limitations were encountered during the process.

## Data Availability

The data described in this Data note can be freely and openly accessed on Figshare (10.6084/m9.figshare.29573585.v1) for Supplementary data file 1, 3–10 [9]. Data file 2 is available on the NCBI GenBank (http://identifiers.org/bioproject:PRJNA1148038) [10]. Please see Table 1 and references [9, 10] for details and links to the data.

## Abbreviations

Xap: *Xanthomonas arboricola* pv. *pruni*
DNA: Deoxyribonucleic acid
NB: Nutrient broth
Bp: Base pair
KEGG: Kyoto Encyclopedia of Genes and Genomes
COG: Clusters of Orthologous Groups
NR: NonRedundant Protein Database databases
GO: Gene Ontology
PHAST: PHAge Search Tool
VFDB: Virulence Factors of Pathogenic Bacteria
ARDB: Antibiotic Resistance Genes Database
PHI: Pathogen Host Interactions
CAZy: Carbohydrate-Active enZYmes Database
